# Supplementary material for: IL-17+ Mast Cell/T Helper Cell Axis in the Early Stages of Acne
Source: Front Immunol. 2021 Sep 28;12:740540. doi: 10.3389/fimmu.2021.740540 (PMC8506309; doi:10.3389/fimmu.2021.740540)
Supplement: Supplementary file 1 [file DataSheet_1.pdf]

Supplementary Material

1 Supplementary Figures and Tables

1.1 Supplementary Figures

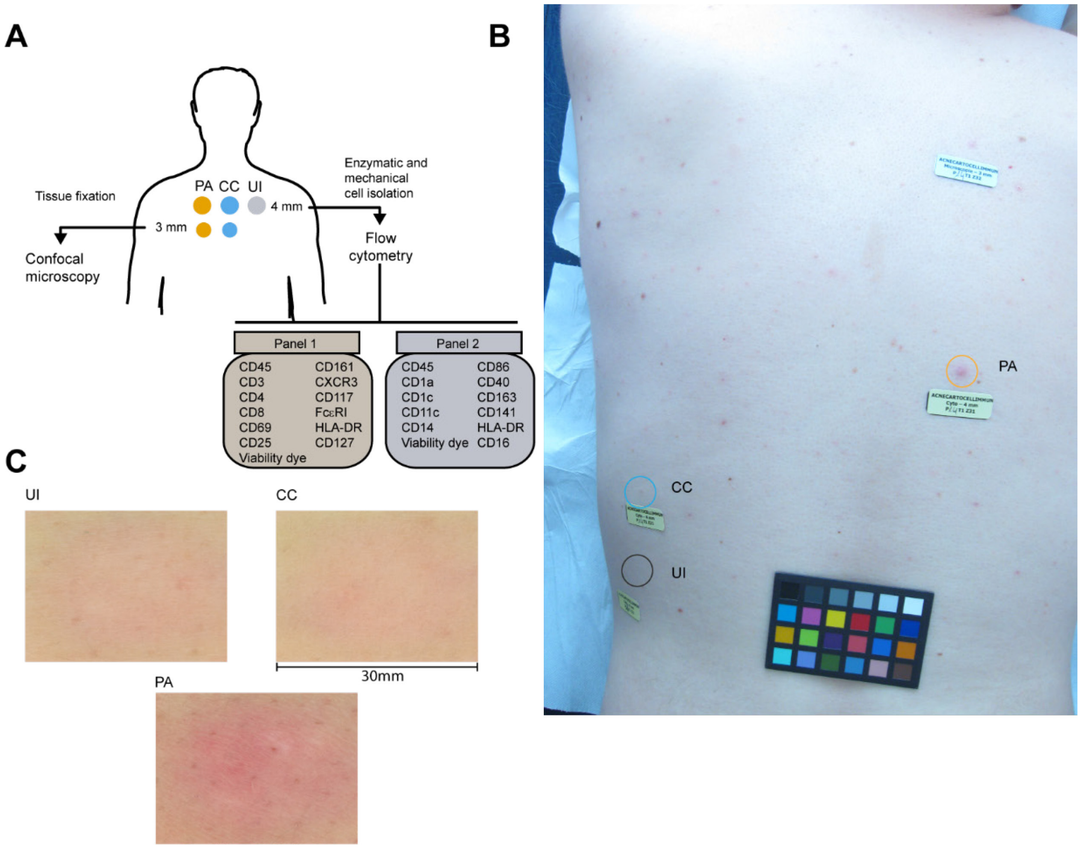

D

Study subject characteristics	
Number of subjects	15
Age, years	20 to 30 - median = 24
Gender	Male
Fitzpatrick skin phenotype	4 subjects phototype II<br>9 subjects phototype III<br>2 subjects phototype IV
Acne severity : IGA score (Investigator's Global assessment)	3 or 4
Inclusion criteria	At least 2 papula and 2 microcomedones on the back and IGA score >= 3 on the back
Facial acne	Mild or absence of facial acne

Supplementary Figure 1. Clinical study characteristics.

(A) Processing workflow for biopsies. The two panels used for immunofluorescence are indicated. **(B-C)** typical example of areas biopsied for UI skin, CC and PA stages (B) and higher magnification, Pixience dermoscope (C). **(D)** Characteristics of the patients involved in this study. CC : closed comedone; PA, papule; UI, uninvolved skin.

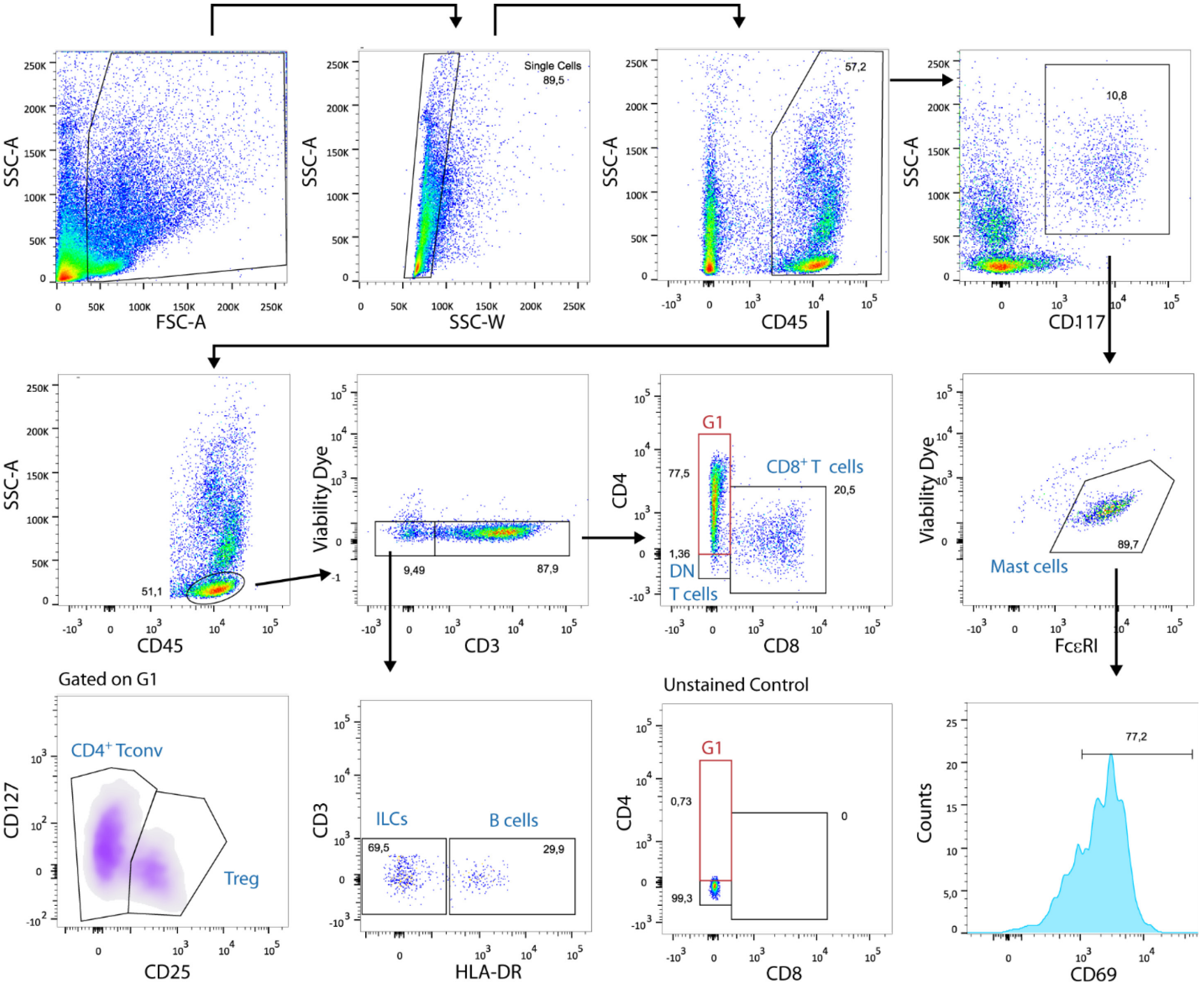

Supplementary Figure 2. Gating strategy used for the analysis of skin cells stained with Abs from panel 1.

Representative dotplots and histograms from a CC biopsy. Targeted populations are indicated in blue. Among $CD4^+$ T cells, regulatory T cells (Treg) were separated from conventional T cells (Tconv) as $CD127^{low}$ $CD25^{high}$ cells. Among $CD3^-$ cells, B cells were identified as $HLA\text{-}DR^{high}$ cells and the remaining cells as innate lymphoid cells (ILCs).

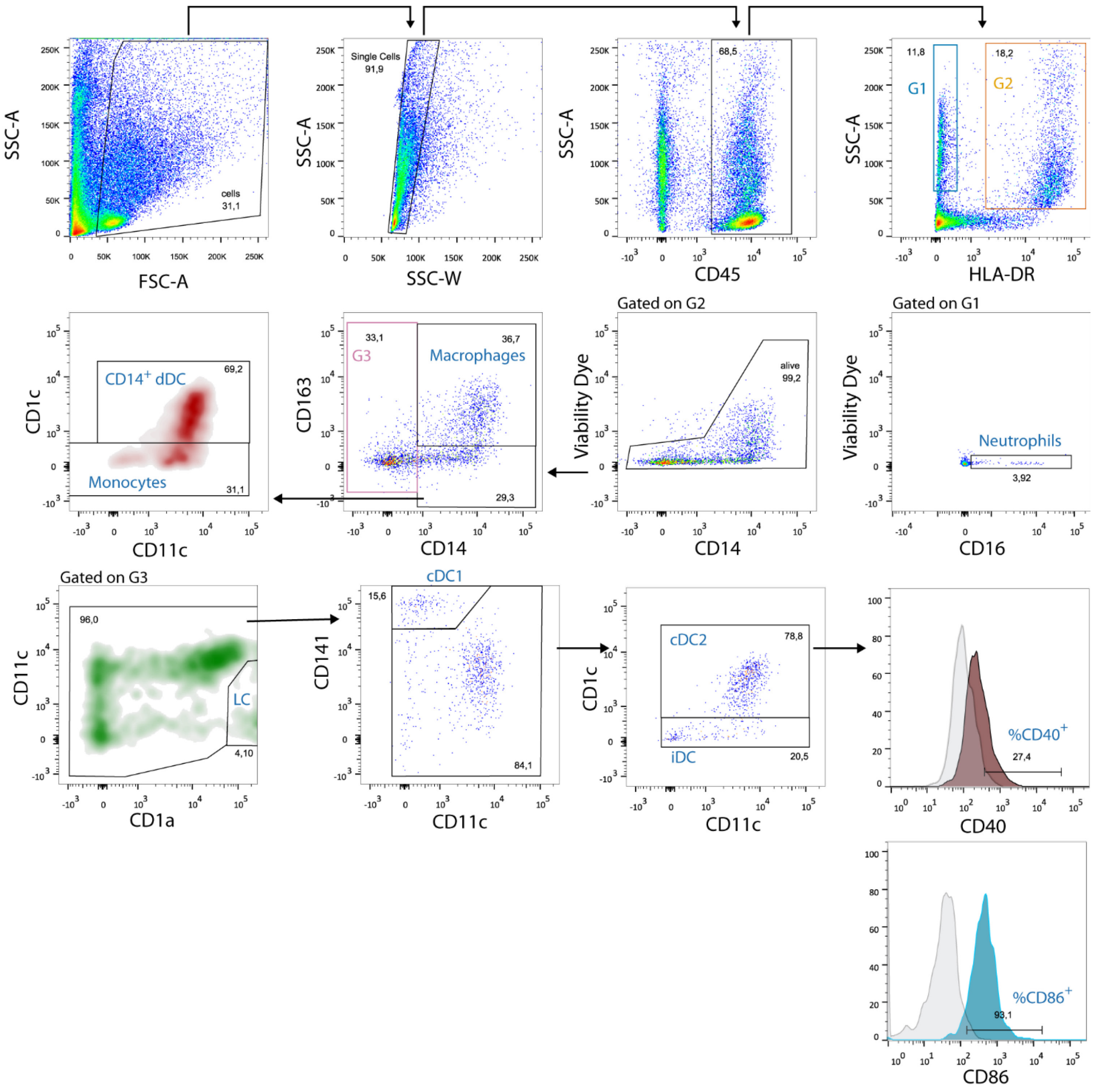

Supplementary Figure 3. Gating strategy used for the analysis of skin cells stained with Abs from panel 2.

Representative dotplots and histograms from CC biopsy. Targeted populations are indicated in blue. Among HLA-DR$^+$ SCC^{high} cells (G2) we did not excluded CD14$^+$ cells that stained for the viability dye because these cells are expected to be macrophages (autofluorescent cells); macrophages were identified as CD14$^+$ CD163$^+$ cells (Zaba et al., 2007), conventional dendritic cells as CD14$^-$ and among CD14$^+$ CD163$^-$ cells we used the CD1c marker to separate monocyte (CD1c$^-$) from CD14$^+$ dermal dendritic cell (CD14$^+$ dDC) (Nestle et al., 1993). These latest were shown to be close to monocytes and a transient population of monocyte-derived macrophages (McGovern et al., 2014). Among the CD14$^-$ DC population, we identified Langerhans cells (CD1a^{high}/CD11c^{low}) (Bigley et al., 2015), conventional DC1s (cDC1, CD141^{high} CD11c^{int}), conventional DC2s (CD1c$^+$, cDC2) and a remaining

population of CD11c$^+$ inflammatory DCs (iDCs) (Clark et al., 2019, Haniffa et al., 2015, Tang-Huau and Segura, 2019).

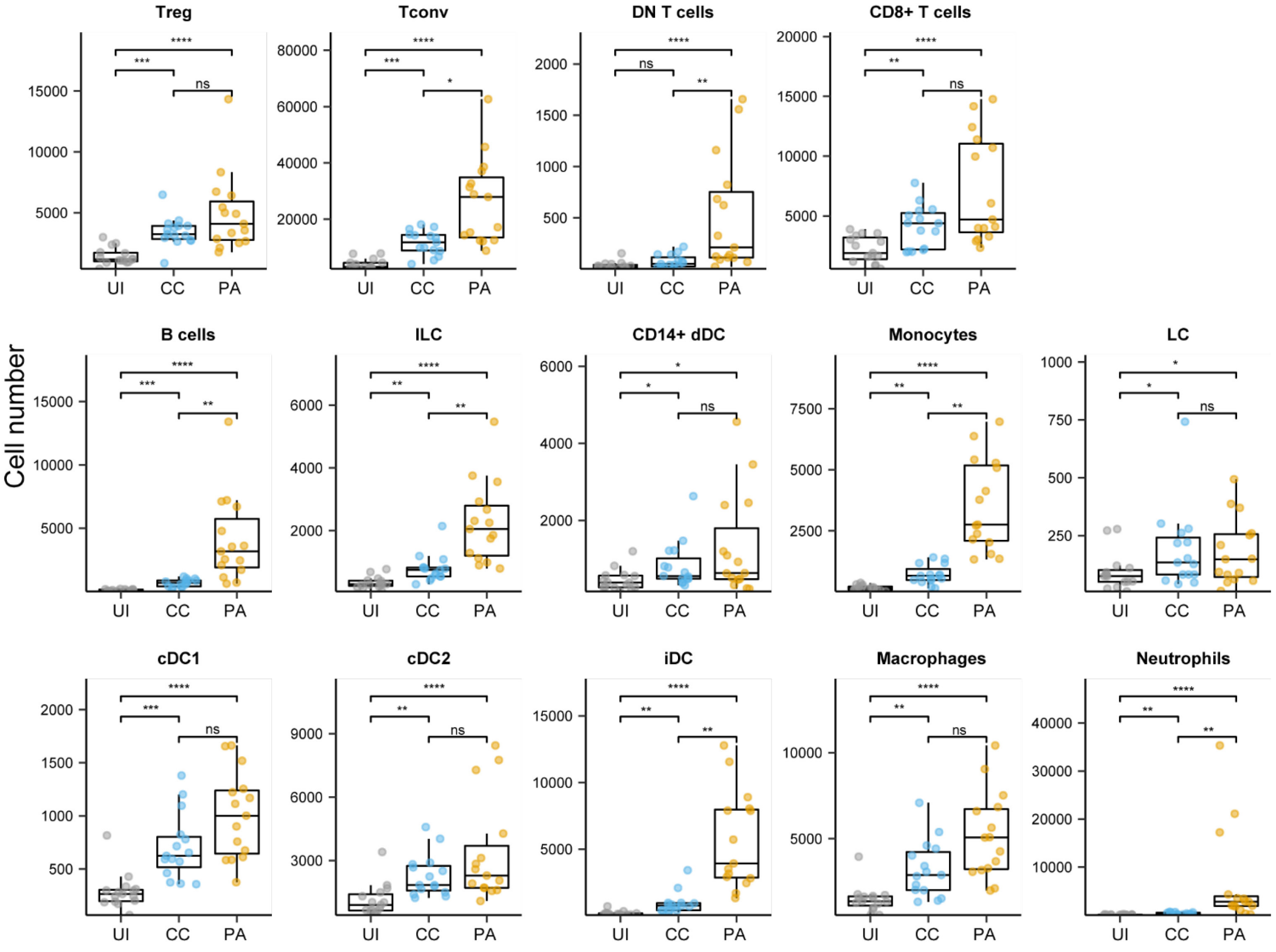

Supplementary Figure 4. Absolute numbers of the main leukocyte populations identified by flow cytometry.

Box and whiskers plot in the style of Tukey. Points represent the values for each of the 15 patients individually. Friedman tests were carried out to compare groups and, if significant, were followed by Dunn's post hoc tests * $p<0.05$; ** $p<0.01$; ***$p<0.001$; **** $p<0.0001$; ns, not significant.

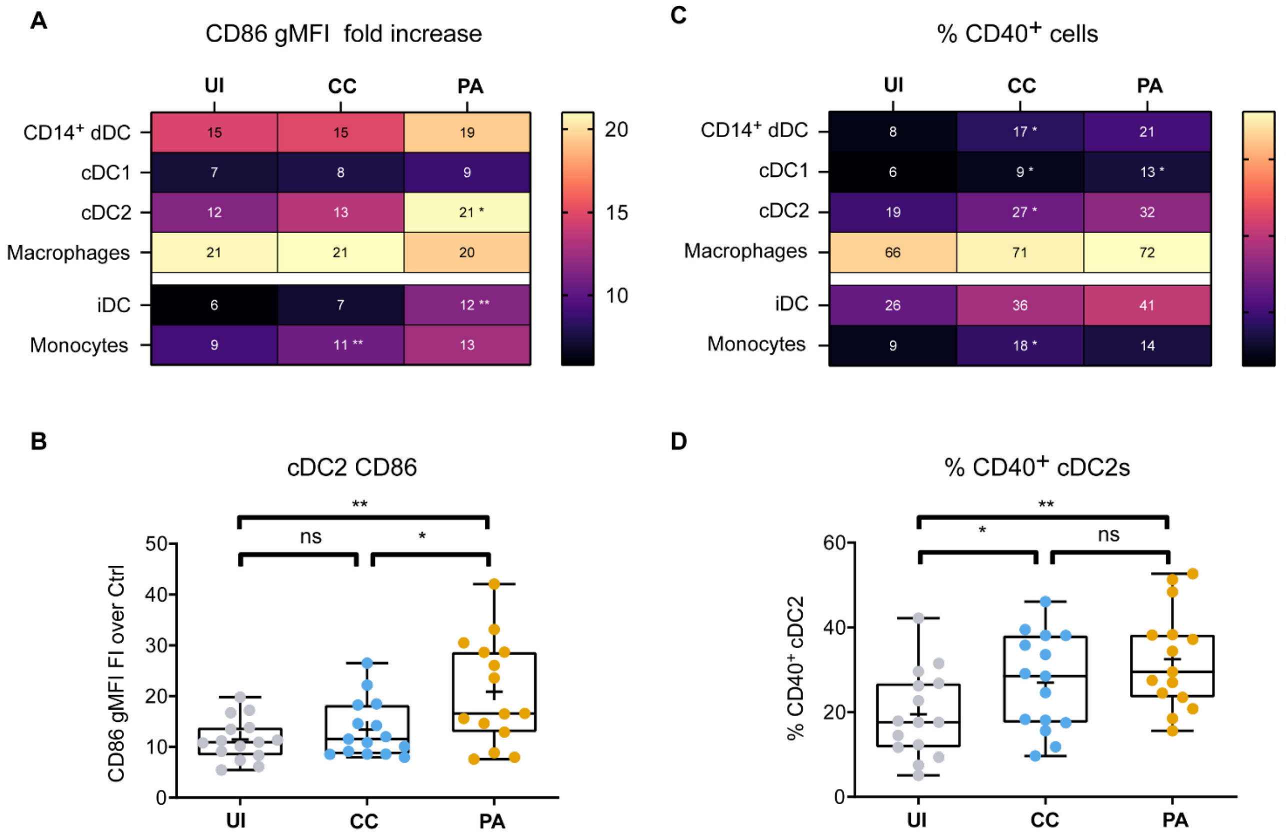

Supplementary Figure 5. cDC2 and CD14⁺dDC showed signs of activation during the early stages of acne.

(A,C) Heatmaps depicting CD86 expression (measured as the fold increase gMFI over the unstained control) (A) and the percentage of $CD40^+$ cells (C) across each APC population, mean values from the 15 patients are indicated in the cells. Statistically significant differences between UI and CC and between CC and PA conditions are indicated in CC and PA cells respectively, * $p<0.05$, **$p<0.01$. **(B,D)** Shown are the cDC2 data from the 15 patients. Box and whiskers represent minimum, 25th percentile, median, 75th percentile and maximum values of CD86 (B) and CD40 (D) expression by cDC2; points represent the individual values for the 15 patients. The cross (+) represents the mean. * $p<0.05$; ** $p<0.01$; ns, not significant (one-way ANOVA and Fisher's least significant difference post hoc test).

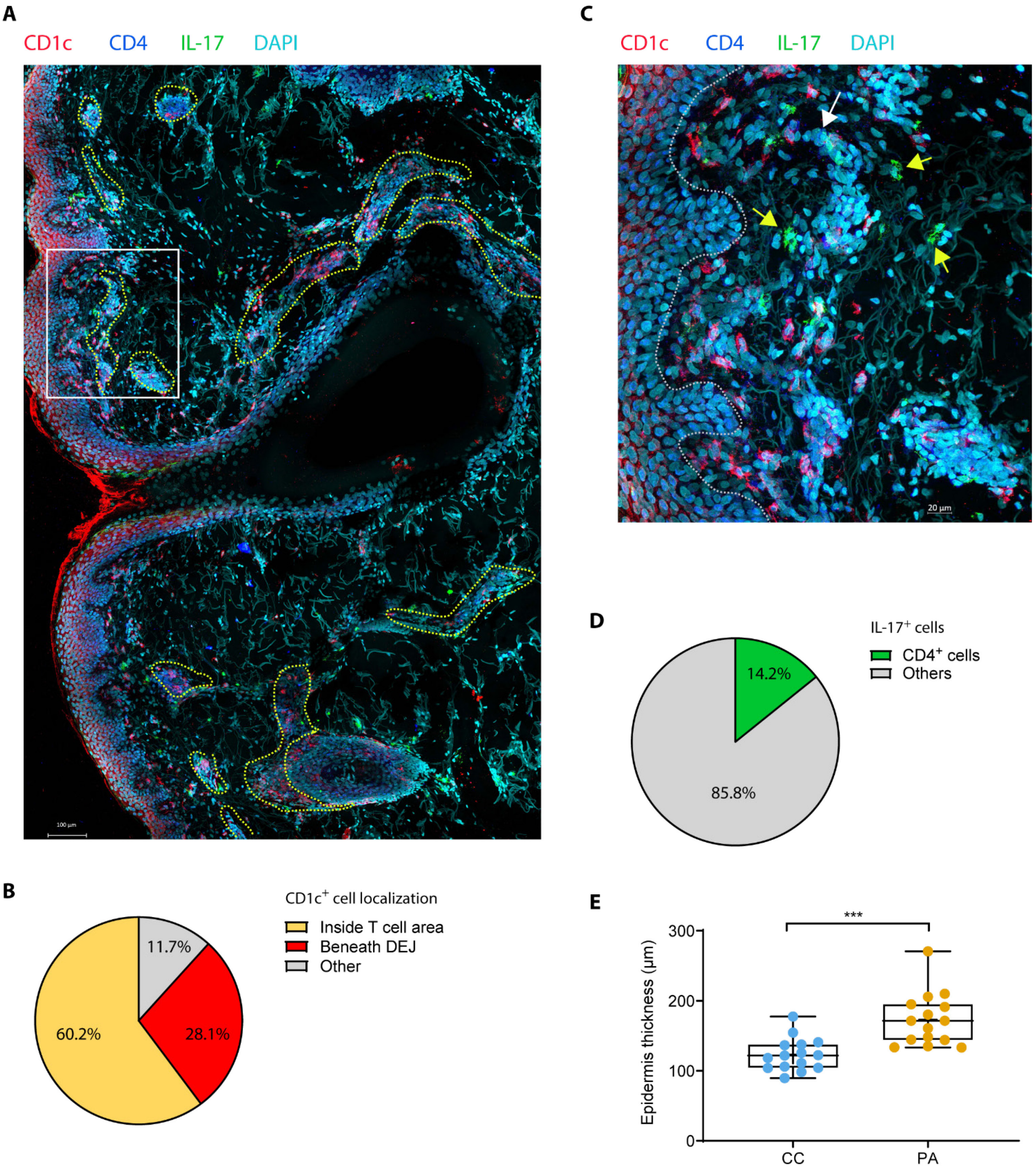

Supplementary Figure 6. CD4^{+} T cells account for only 14% of IL-17-producing cells in CC biopsies.

(A) Representative confocal laser scanning microscopy tile scan from an entire CC biopsy section presented in maximum intensity projection of a z-stack series. Perifollicular and perivascular regions are delineated with yellow dashed lines. Enrichment of CD1c^{+} cells beneath the dermal-epidermal

junction (DEJ) and inside T cell-rich areas is observed. **(B)** Quantification of $CD1c^+$ cell localization (n=5 CC biopsies). **(C)** Magnification of a T cell area showing $IL17^+$ $CD4^+$ T cell (white arrow) and IL-17^+ non-$CD4^+$ unidentified cells (yellow arrows). **(D)** Quantification of IL-17^+ $CD4^+$ cells (3 CC biopsies were analyzed, n=218 IL-17^+ cells). **(E)** Epidermis thickness measurement (n=15 patients). Each point represents a patient (mean of 6 measurements taken at regular intervals along the epidermis with Zen software). ***, $p<0.001$ (Student's *t*-test).

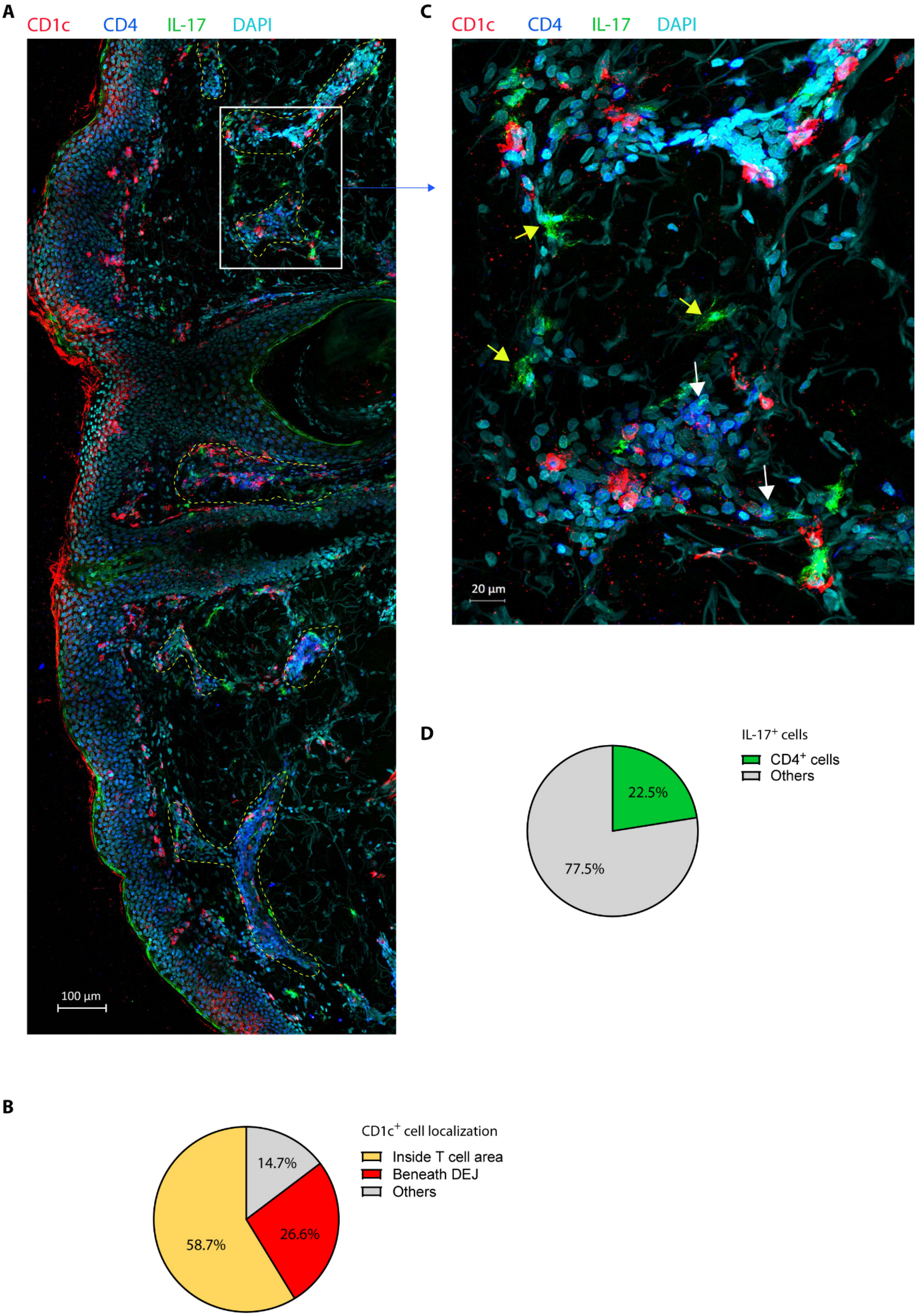
A
CD1c CD4 IL-17 DAPI
100 µm
C
CD1c CD4 IL-17 DAPI
20 µm
D
IL-17+ cells
CD4+ cells
Others
22.5%
77.5%
B
CD1c+ cell localization
Inside T cell area
Beneath DEJ
Others
14.7%
26.6%
58.7%

Supplementary Figure 7. $CD4^+$ T cells account for less than 23% of IL-17-producing cells in PA biopsies.

(A) Representative confocal laser scanning microscopy tile scan from an entire PA biopsy section, presented in maximum intensity projection of a z-stack series. Perifollicular and perivascular regions are delineated with yellow dashed lines. **(B)** Quantification of $CD1c^+$ cell localization (n=5 PA biopsies). **(C)** Magnification of a T cell area showing IL-17^+ $CD4^+$ T cell (white arrows) and IL-17^+ non-$CD4^+$ unidentified cells (yellow arrows). **(D)** Quantification of IL-17^+ $CD4^+$ cells (3 PA biopsies were analyzed, n=298 IL-17^+ cells).

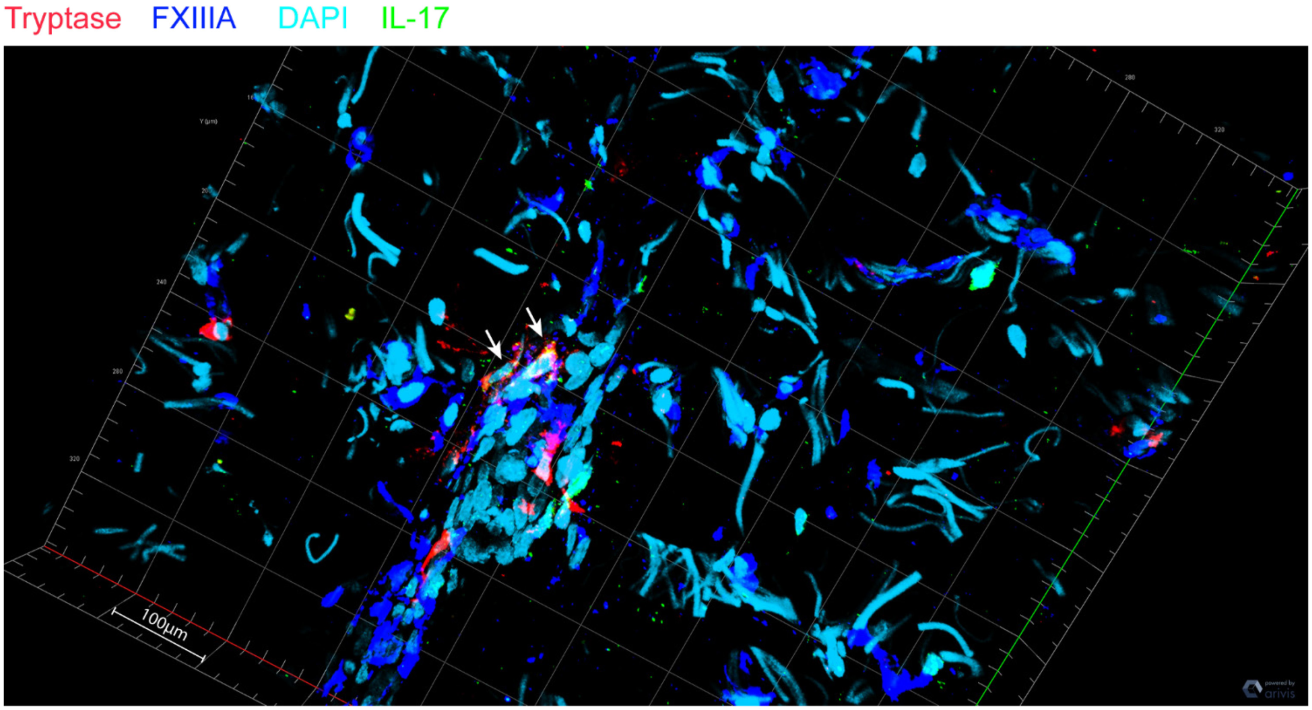

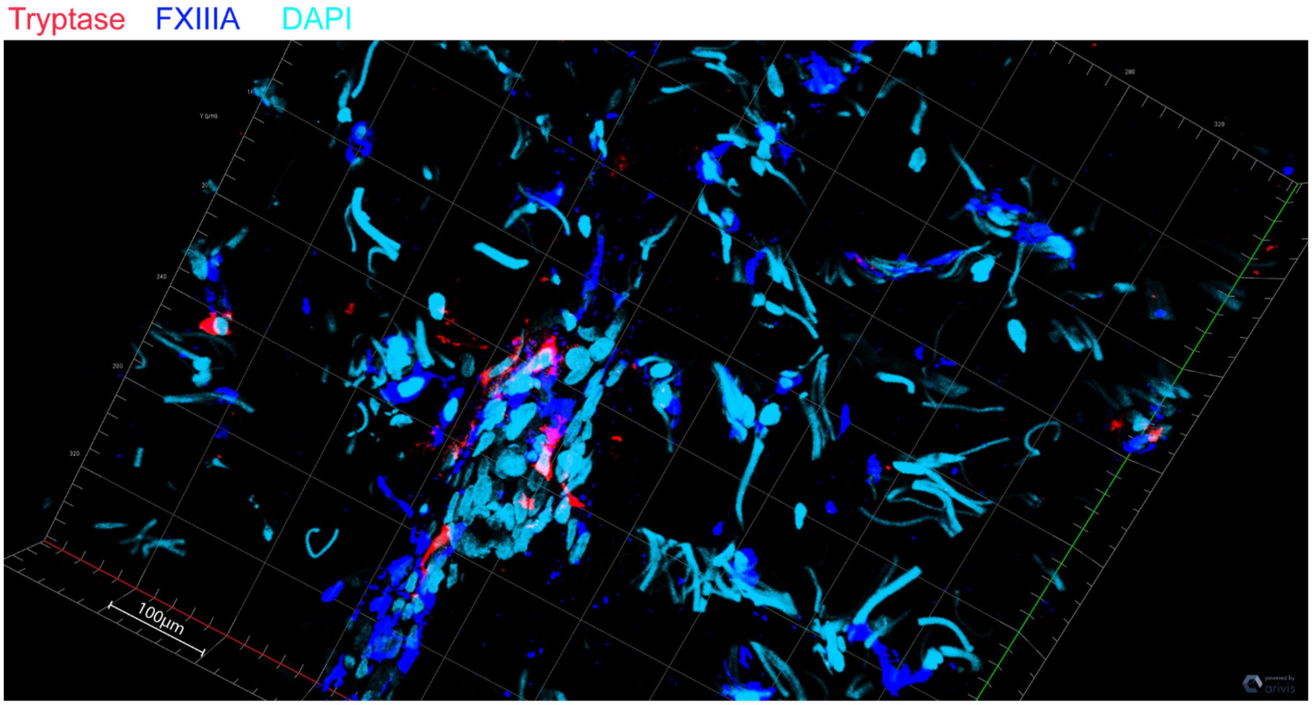

Supplementary Figure 8. Macrophages did not produce IL-17 in acne biopsies analyzed.

3D reconstruction from laser confocal scanning z-stack depicting a perivascular area in the dermis of a PA biopsy. Representative image showing several FXIIIA^{+} (blue) macrophages staining negative for IL-17 (green).

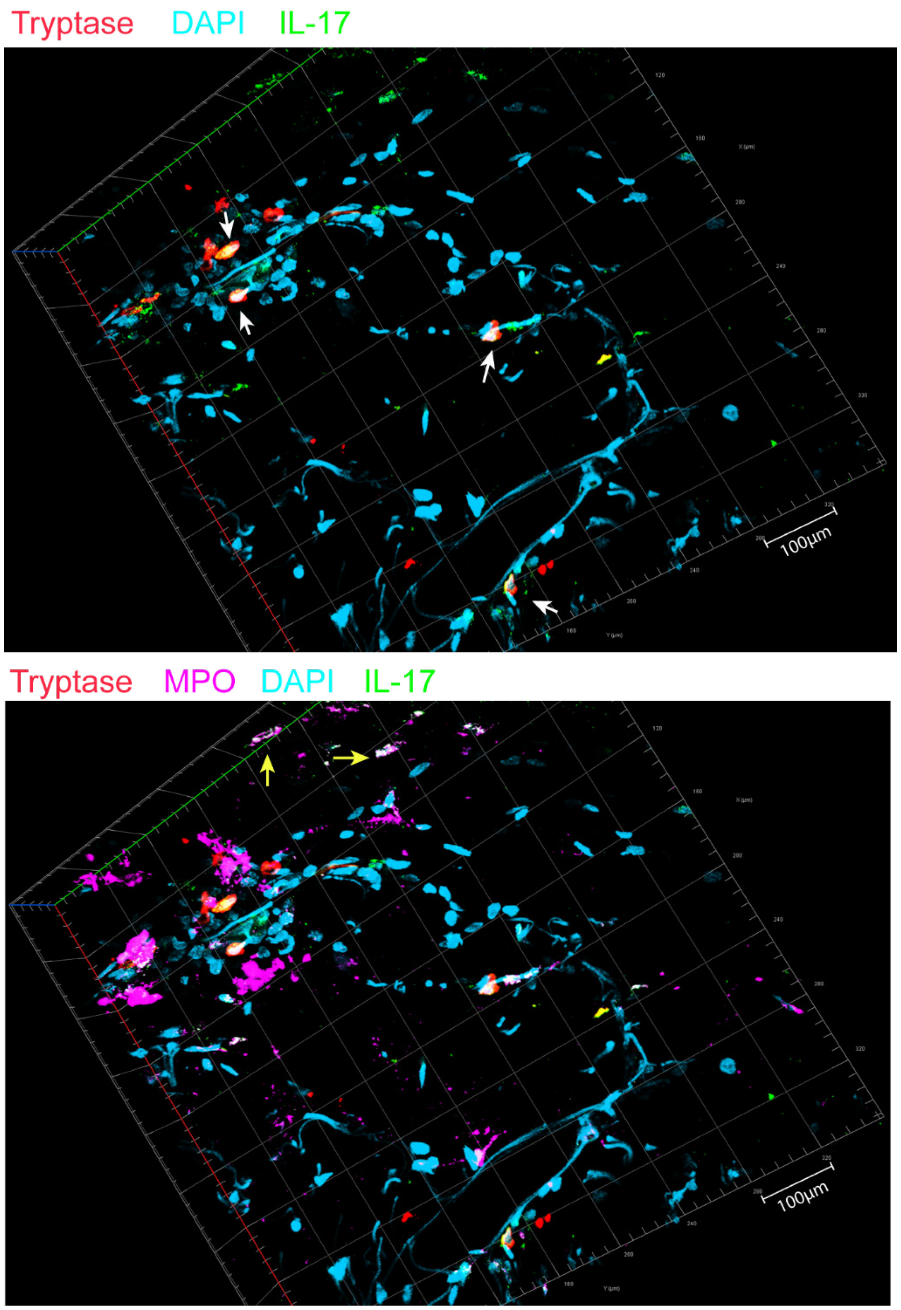

Supplementary Figure 9. A fraction of the neutrophils stained IL-17$^+$ in PA biopsies.

3D reconstruction from laser confocal scanning z-stack depicting a perivascular area in the dermis of a PA biopsy. Representative image showing some MPO$^+$ (pink) neutrophils (yellow arrows) staining positive for IL-17 (green) and some IL-17$^+$ tryptase$^+$ mast cells (white arrows).

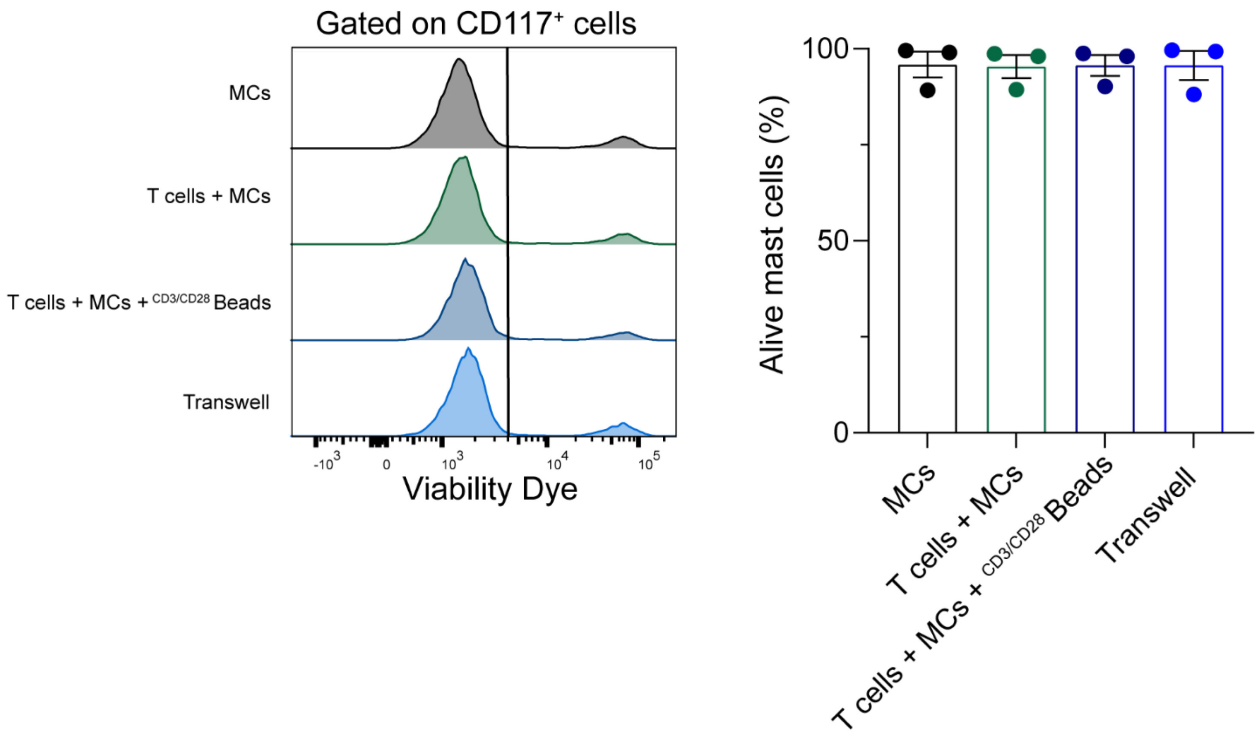

Supplementary Figure 10. Mast cell viability is not altered by the 48h coculture.

MCs cocultured alone or with resting or activated $CD4^+$ T cells for 48h were analyzed by FACS for cell sorting. MC viability was assessed by viability dye staining. Tranwell indicates that MCs and activated $CD4^+$ T cells (via anti-CD3/CD28 coated beads) were separated in coculture with Transwell system.

Donkey anti-rabbit IgG
Donkey anti-goat igG
Donkey anti-mouse IgG DAPI

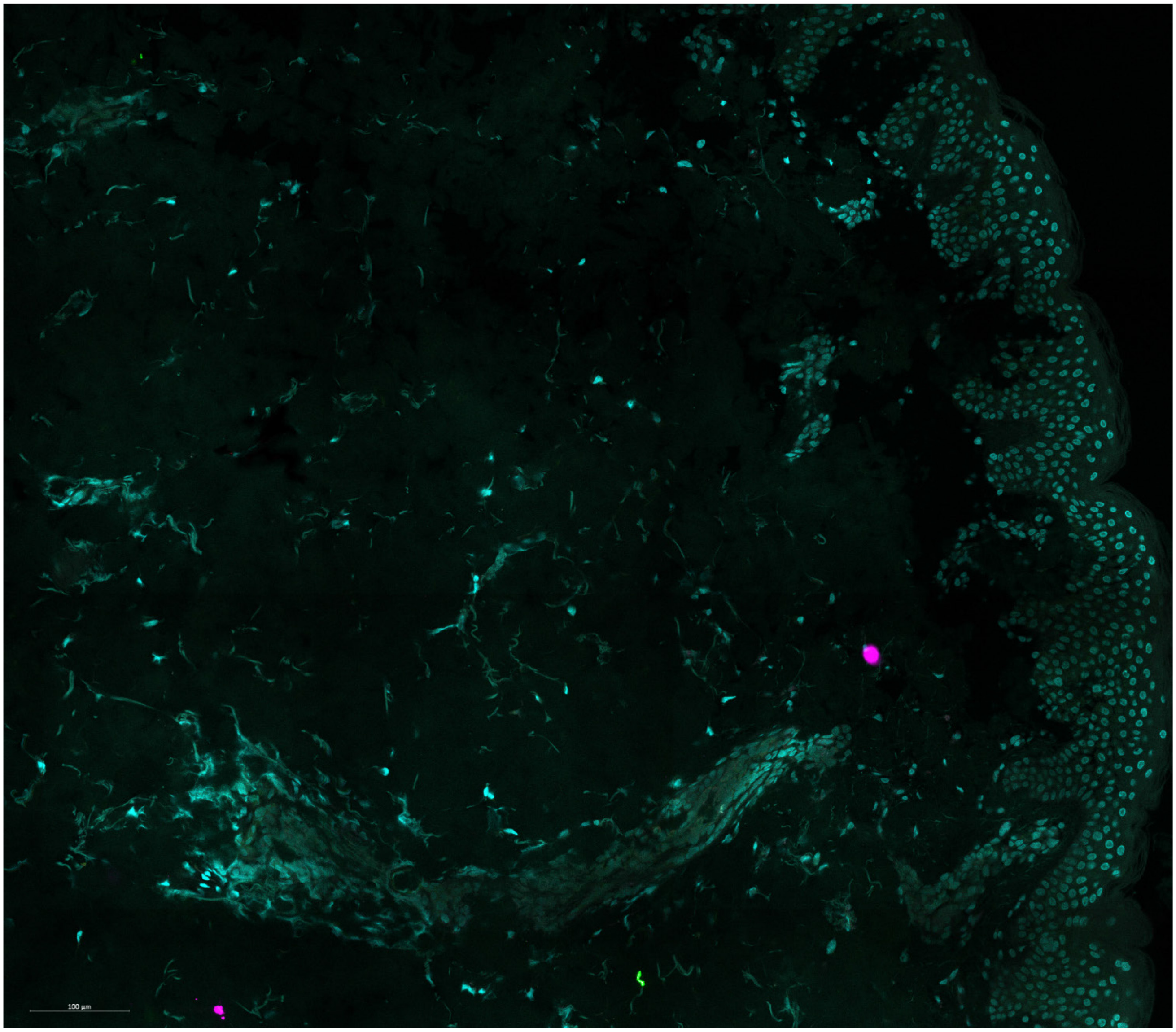

Supplementary Figure 11. Example of staining control in confocal microscopy.

Image obtained using staining procedure with secondary Abs alone (Donkey anti-goat IgG-Alexa-488, Donkey anti rabbit IgG-Alexa-647 and Donkey-anti-mouse-Alexa-555) allowing the confocal settings for image acquisition.

1.2 Supplementary Table

Antibodies for direct immunofluorescence (flow cytometry)				
Antigen	Fluorophore	Clone	Panel	Manufacturer
CD3	BV786	SK7	P1	BD
CD4	PE-cy7	SK3	P1	BD
CD8	PerCP-Cy5.5	RPA-T8	P1	BD
CD25	PE-CF594	M-A251	P1	BD
CD69	BV711	FN50	P1	BD
CD117	APC-R700	YB5.B8	P1	BD
CD127	BB515	HIL-7R-M21	P1	BD
CD161	BV650	DX12	P1	BD
CXCR3	APC	1C6/CXCR3	P1	BD
HLA-DR	PE	G46-6	P1 & P2	BD
FcεRI	e450	AER-37 (CRA1)	P1	eBiosciences
CD1a	APC	HI149	P2	BD
CD1c	BB515	F10/21A3	P2	BD
CD14	PE-CF594	MfP9	P2	BD
CD40	BV421	5C3	P2	BD
CD45	APC-H7	2D1	P1 & P2	BD
CD86	BV711	2331 (FUN-1)	P2	BD
CD163	BV650	GHI/61	P2	BD

CD11c	BV785	3.9	P2	Biolegend
CD16	AF700	3G8	P2	Biolegend
CD141	PE-Cy™7	M80	P2	Biolegend
Primary antibodies for indirect immunofluorescence (microscopy)				
Agntigen	Isotype	Clone		Manufacturer
Tryptase	Mouse IgG1	AA1		Millipore
IL-17	Goat polyclonal			R&D systems
CD1c	Mouse IgG1	5B8		Abcam
CD4	Rabbit polyclonal			Sigma-Aldrich
CD3	Rabbit polyclonal			Dako
MPO	Rabbit polyclonal			Dako
FXIIIA	Rabbit polyclonal			Thermo scientific

Table S1. Antibodies used in immunofluorescence.
